# Amphetamine Dependence and Co-Morbid Alcohol Abuse: Associations to Brain Cortical Thickness

**DOI:** 10.1186/1471-2210-10-5

**Published:** 2010-05-20

**Authors:** Glenn Lawyer, Petr S Bjerkan, Anders Hammarberg, Nitya Jayaram-Lindström, Johan Franck, Ingrid Agartz

**Affiliations:** 1 Department of Psychiatry, University of Oslo, Oslo, Norway; 2 Department of Computational Biology, Max Planck Institute for Informatics, Saarbrücken, Germany; 3 Department of Clinical Neuroscience, Karolinska Institutet, Stockholm, Sweden; 4 Department of Psychiatric Research, Diakonhjemmet Hospital, Oslo, Norway

## Abstract

**Background:**

Long-term amphetamine and methamphetamine dependence has been linked to cerebral blood perfusion, metabolic, and white matter abnormalities. Several studies have linked methamphetamine abuse to cortical grey matter reduction, though with divergent findings. Few publications investigate unmethylated amphetamine's potential effects on cortical grey matter. This work investigated if amphetamine dependent patients showed reduced cortical grey matter thickness. Subjects were 40 amphetamine dependent subjects and 40 healthy controls. While all subjects were recruited to be free of alcohol dependence, structured clinical interviews revealed significant patterns of alcohol use in the patients. Structural magnetic resonance brain images were obtained from the subjects using a 1.5 Tesla GE Signa machine. Brain cortical thickness was measured with submillimeter precision at multiple finely spaced cortical locations using semi-automated post-processing (FreeSurfer). Contrast analysis of a general linear model was used to test for differences between the two groups at each cortical location. In addition to contrasting patients with controls, a number of analyses sought to identify possible confounding effects from alcohol.

**Results:**

No significant cortical thickness differences were observed between the full patient group and controls, nor between non-drinking patients and controls. Patients with a history of co-morbid heavy alcohol use (n = 29) showed reductions in the superior-frontal right hemisphere and pre-central left hemisphere when compared to healthy controls (n = 40).

**Conclusions:**

Amphetamine usage was associated with reduced cortical thickness only in patients co-morbid for heavy alcohol use. Since cortical thickness is but one measure of brain structure and does not capture brain function, further studies of brain structure and function in amphetamine dependence are warranted.

## Background

Amphetamines are central nervous system stimulants. They are among the most commonly abused illicit drugs after cannabis [1]. Mounting evidence shows that illicit users expose themselves to substantial risk of brain injury in frontal and subcortical regions. Blood perfusion abnormalities are well established [2-5]. Metabolic abnormalities are widely reported, including reduction in dopamine transporters in the striatum [6-8] and prefrontal cortex [9]; reduced levels of neuronal markers (N-acetylaspartate) in frontal white matter and increased myo-inositol and choline-containing compounds in frontal grey matter [10]; and altered cerebral glucose metabolism [11,12]. A recent review of evidence for structural brain abnormalities in illicit amphetamine users reported white matter abnormalities including volume reductions, disturbed fractional anisotropy measures, and increased deep and periventricular white matter hyperintensities (WMH) [13]. These findings were echoed in a later publication which again found lower fractional anisotropy in right frontal white matter and other white matter disturbances in the basal ganglia of methamphetamine using patients using diffusion tensor imaging [14].

The combination of perfusion, metabolic, and white matter abnormalities provide biological mechanisms which could potentially result in primary and/or secondary detrimental effects on cortical grey matter. Metabolic abnormalities may lead to apoptosis, or loss of glial cell volume with concurrent reduction in cortical thickness. Infarcts arising from blood perfusion defects could result in cortical tissue loss through Wallerian degeneration. The lower fractional anisotropy noted by [14] was interpreted as evidence for axonal injury. It has been observed in Alzheimers patients that blood perfusion abnormalities are evident before cortical loss [15]. The Hawaii Aging with HIV cohort study has provided evidence linking WMHs with cortical atrophy [16].

Behavioral evidence also suggests that amphetamine usage is associated with abnormalities in frontal regions. One characteristic of addictive behavior is preference for small immediate rewards over larger but delayed rewards. A comparison of 19 recently abstinent methamphetamine using subjects with 17 healthy controls found that the patients showed less activation than controls in the anterior cingulate cortex, dorsolateral prefrontal cortex, and subcortical regions in a choice of reward task [17]. Addiction can also be characterized by deficit in behavior control and/or modification. A study comparing 12 methamphetamine dependent patients with 16 healthy controls found that the patients exhibited reduced activation in the right prefrontal cortex during a behavior control task [18]. Neither study, however, was able to elucidate if the observed cognitive and neural activation differences were a predisposing factor or a consequence of the addictive behavior.

Yet evidence of amphetamine related cortical grey matter damage in the substance dependant population remains equivocal. Berman et al.'s (2008) review [13] listed cortical grey matter results from four studies: two compared methamphetamine using and control subjects, two were polydrug studies which included methamphetamine users. The first methamphetamine study used cortical surface maps to compare localized grey matter density estimates in 22 methamphetamine using subjects compared to 21 healthy controls. It found reductions in the cingulate, limbic, and paralimbic cortices, but not in the lateral cortical surfaces [19]. The second employed voxel-based morphometry to compare grey matter density in 29 methamphetamine using subjects compared to 20 healthy controls. It reported reduced grey matter density in the right middle frontal gyrus [20]. One polydrug study compared manually traced regions of interest in 9 methamphetamine using, 10 cocaine using, and 16 control subjects. It reported smaller temporal grey matter volumes in both amphetamine and cocaine using compared to control men, with no overall difference between the stimulant using groups in the estimated grey matter volumes [21]. The other investigated 16 intravenous drug users, nine of whom abused methamphetamine along with other drugs, and found no reductions in either whole brain or frontal lobe grey matter volumes compared to 16 matched controls, though white matter reductions were noticed in both patient groups [22].

None of the studies in the review investigated grey matter changes in unmethylated amphetamines (i.e. racemic or dextroamphetamine). In Northern Europe, however, the vast majority of amphetamine abused is unmethylated [1]. Members of our group has also observed this trend in a clinical study based on bi-weekly urine toxicology tests of eighty patients at the Addiction Centre, Stockholm [23].

Separating amphetamine effects from other drug related effects on brain cortical thickness is made difficult as a diagnosis of amphetamine abuse is frequently concurrent with abuse of other drugs such as alcohol or nicotine, with one study showing co-morbidity levels in excess of 90% for both substances [24]. Alcohol abuse can lead to measurable cortical grey matter reductions [25-28], which may be exacerbated by chronic smoking [29].

The objective of the present study was to ascertain if amphetamine related brain cortical thickness deficits would be observed in a comparatively large real-world patient sample. Much of the evidence for amphetamine-related brain injury, including the above-cited work on blood perfusion [3,5], metabolism [9,10], and white matter morphology [13,14], suggests cortical injury would occur in frontal regions. As this suggestion is further supported by behavioral evidence [17,18], we suspected that that amphetamine use might also associate with reduced grey matter thickness in frontal and prefrontal regions. To the best of our knowledge, this is the first study of cortical thickness alterations in amphetamine using, as opposed to methamphetamine using, subjects.

## Results

**Figure 1:**
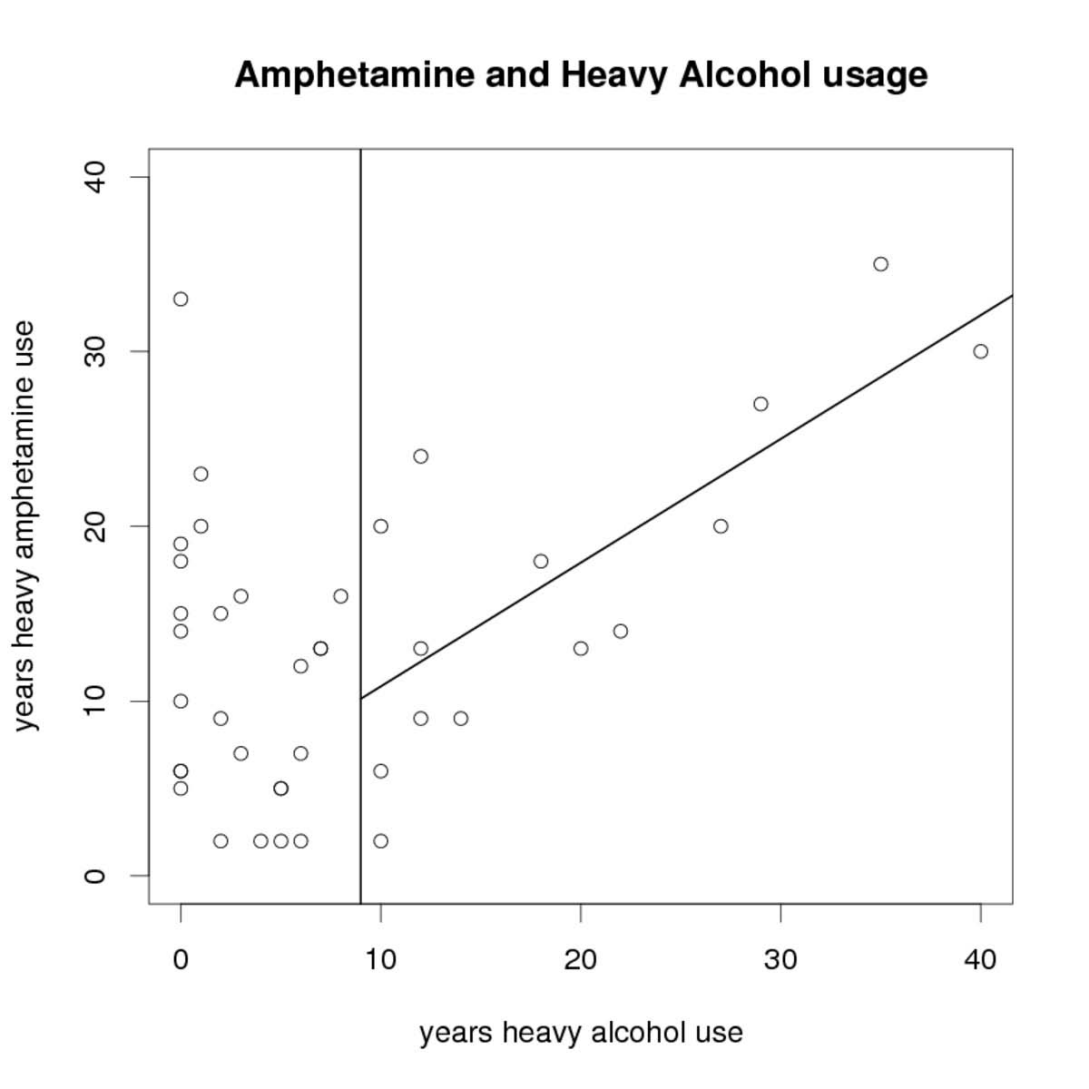
**Years of heavy amphetamine use and heavy alcohol use**. The correlation between years of heavy amphetamine use and heavy alcohol use in the current patient sample was significant in patients with 10 or more years heavy alcohol use, but not in patients with less. The black slanted line shows the line of best fit for this subset. The vertical line marks patients with 10 or more years of heavy alcohol use.

Cortical regions showing amphetamine related thickness differences were identified by repeatedly testing for significant group differences at numerous finely spaced cortical locations. The test was a contrast analysis of a general linear model, a generalization of an ANOVA test. The patients had been recruited for not having a diagnosis of alcohol dependence and none met DSM diagnostic criteria for alcohol dependence. Nonetheless, structured interviews found that 31 of the 40 patients had a history of heavy alcohol consumption (see Figure 1). In order to identify effects attributable to the confounding effect of alcohol usage, the patients were subdivided into those with 0-1 years of heavy alcohol use (*A*_0-1_, n = 11), and those with more than 1 year of heavy alcohol use (*A*_>1_, n = 29). The following four contrasts were tested:

a) all patients (*A*_0-1 _+ *A*_>1_) (n = 40) vs controls (n = 40);

b) *A*_0-1 _patients (n = 11) vs controls;

c) *A*_>1 _patients (n = 29) vs controls;

d) *A*_0-1 _vs *A*_>1 _patients.

Contrast *c *was then repeated with patients restricted to those with 10 or more years of heavy alcohol use (*A*_≥10_, n = 14). At a reviewer's suggestion, contrast *c *was also run with the original subject group (*A*_>1_, n = 29) but with years of heavy alcohol use added to the model as a covariate. This is analogous to a one-way ANOVA containing years of heavy alcohol use.

**Figure 2:**
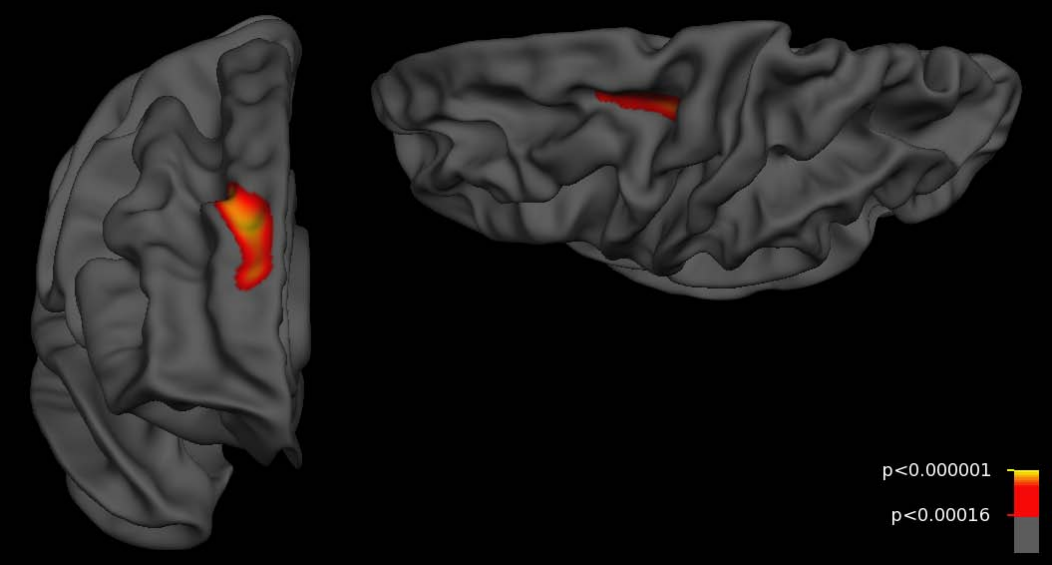
**Cortical thickness differences between controls and alcohol-using amphetamine dependent subjects**. Controls showed thicker cortex than patients with more than one year of heavy alcohol use (*A*_≥1 _*vs control*) in superior-frontal regions, covering more areas in the right than left hemisphere. Views are rostral right hemisphere and dorsal left hemisphere. Colored regions indicate *p*-values less than 0.00016, a threshold for which the expected number of false positives is under 5%. Colors are coded by (-log_10_), with red indicating *p *= 0.00016 and yellow indicating *p *< 0.000001.

**Figure 3:**
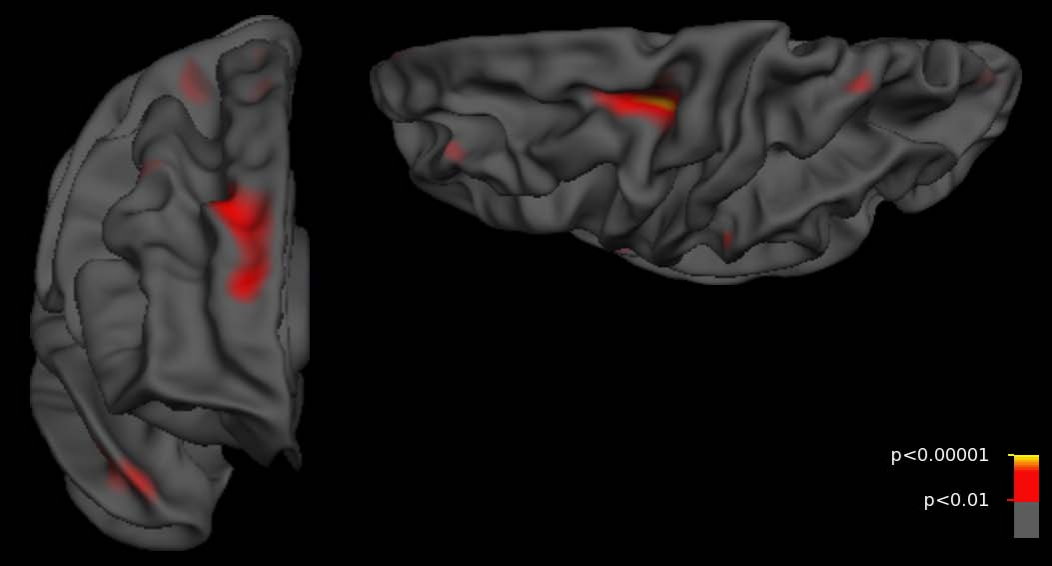
**Cortical thickness differences between controls and long-term alcohol using amphetamine dependent subjects**. Controls showed trends towards thicker cortex than patients with ten or more years of heavy alcohol use (*A*_≥10 _*vs control*) in superior-frontal regions. Views are rostral right hemisphere and dorsal left hemisphere. *P*-values are uncorrected. No results survive after controlling the FDR at a 5% level. Colors are coded by (-log_10_), with red indicating *p *= 0.01 and yellow indicating *p *< 0.00001.

The results of each of the contrast analyses were as follows:

a) All amphetamine users compared to healthy control subjects (*A*_0-1 _+ *A*_>1 _*vs*. *control*) showed no significant differences for any cortical region.

b) Patients with limited heavy alcohol use compared to healthy controls (*A*_0-1 _*vs. control*) showed no significant differences for any cortical region.

c) Patients with more than 1 year of heavy alcohol use compared to healthy controls (*A*_>1 _*vs. control*) showed thinner cortex in right hemisphere superior-frontal regions and left hemisphere pre-central regions in comparison to healthy controls. Significantly thinner cortex was signaled by *p*-values less than 0.00016, a threshold with an expected false discovery rate under 5%. When the analysis was restricted to patients with 10 or more years of heavy alcohol use (*A*_≥10 _*vs. control*), the findings were similar but not strong enough to survive FDR correction at the 5% level. This could be due to a loss of power in the restricted subject sample. Results from these two contrasts are shown in Figures 2 and 3. When the model included years of heavy alcohol use, no results remained significant after FDR correction.

d) Patients with limited heavy alcohol use compared with patients with more than 1 year of heavy alcohol use *(A*_0-1 _*vs. A*_>1_) showed no significant differences for any cortical region.

A final set of analyses were conducted to further help distinguish between amphetamine-related and alcohol-related differences. This set restricted itself to patients with less than 10 years of heavy alcohol use (*A*_<10_, n = 26), who were divided into those with long-term amphetamine use (more than 10 years, n = 13) and those with short-term amphetamine use (10 years or less, n = 13). Two contrasts were tested:

e) long-term users vs short-term users, and

f) long-term users vs healthy controls (n = 40).

In these final analyses, age was controlled for but not years of amphetamine use. No significant differences were observed in either of these two analyses.

## Discussion

This Swedish sample of amphetamine dependent patients showed no convincing evidence of reduced cortical thickness except in conjunction with heavy alcohol consumption. Significant findings were limited to thinner cortex in right superior-frontal regions and the left pre-central gyrus when heavy alcohol-using amphetamine subjects were compared to controls. These disappeared when the model controlled for years of heavy alcohol usage. No significant difference was found between the full patient group and controls. This suggests that the findings reflect the effects of alcohol. Alcohol has been shown to negatively affect frontal brain regions in a number of studies. Alcohol-related brain volume shrinkage was exaggerated in the prefrontal cortex [28]. Another research lab observed that alcohol-related reductions in brain tissue were seen primarily in prefrontal and parietal regions [25]. Selective neuronal loss in superior frontal association cortex in chronic alcoholics was reported by [26].

The lack of difference between patients with limited and long-term heavy alcohol use (contrast d, *A*_0-1 _vs *A*_>1_), suggests the possibility of an interaction effect. Keeping in mind, however, that 90% of the current patients reported use of nicotine at the time of scan, and that Gazdzinski et al. (2005) [29] found that chronic cigarette smoking may exacerbate regional chronic alcohol-induced brain injury, it is not possible to say if any speculated interaction was with amphetamine or nicotine.

The findings in the right hemisphere, here only found in conjunction with heavy alcohol use, correspond to the results reported in Kim et al.'s (2006) study of methamphetamine users [20]. This suggests a possibility of increased neurotoxicity in methamphetamine compared to amphetamine. Thompson et al.'s 2004 investigation of methamphetamine users, however, did not find evidence of grey matter loss in the frontal lobe [19], but did find grey matter deficit in cingulate, limbic, and paralimbic regions.

Screening for HCV was not present at the beginning of this study as awareness that HCV may cause brain dysfunction has arisen only in the last few years [30]. HCV prevalence was high in this subject population, as is typical for illicit intravenous drug users. Mounting evidence suggests that HCV plays a role in many of the neurological alterations observed in amphetamine dependent subjects, such as the reduced dopamine transporters and N-acetylaspartate levels, along with increases in choline-containing compounds and alterations in cerebral glucose mechanisms [30,31]. Conventional MR imaging, however, as used in the current study, was found to be normal in HCV positive subjects compared to controls [30]. Thus while it is likely that HCV infections have negative neurological effects on our patients, little evidence suggests that HCV has directly influenced the negative findings in this study of cortical thickness.

The methamphetamine using patient was not excluded as the clinical interviews and urine toxology tests showed that patient also was using a racemic mixture. Methamphetamine usage is a recent phenomenon in the Nordic region [1]. It thus appeared that the patient was beginning to switch to methamphetamine, but that his 5 year career had included significant amounts of racemic amphetamine usage.

The present study's exclusion criteria were designed to eliminate cortical thinning due to use of illicit substances other than amphetamine or infection with HIV. Structured clinical interviews confirmed that polydrug use in the current subjects was limited. Infection with HIV was assessed only by an interview question. We did not exclude for the presence of symptoms fulfilling a DSM-IV diagnosis of attention-deficit hyperactivity disorder (ADHD). The analysis did not control for years of education, though this variable was lower in the patients compared to the controls. Since, however, all of these factors are typically associated with thinner cortex, and no difference was seen in our patents, it seems unlikely that any of these potential confounds explain the lack of non-alcohol associated differences.

The main analysis divided patients into those with one or less year of heavy alcohol use and those with more than one year of heavy alcohol use. This was necessitated by the low subject numbers reporting no history of heavy alcohol use. As alcohol-related brain tissue loss depends on length of use [28], a one year cutoff was chosen. Follow-up analysis divided subjects using 10 or more years of heavy alcohol use as a cutoff. This level, while supported by a correlation analysis, remains arbitrary. It is likely that the correlation analysis would have produced similar results had the threshold been 9, or 11 years, nor should any correlation reported here be considered representative of the general population.

Strengths of the present study were the comparatively large well-characterized subject material and the method to examine cerebral cortex thickness throughout the entire cortical mantle with submillimeter precision.

## Conclusions

No evidence was found for a relationship between amphetamine usage and cortical grey matter thickness reductions, except in the presence of co-morbid heavy alcohol usage. We note that cortical thickness is only one measure of brain anatomy. Amphetamine and methamphetamine neurotoxicity has been demonstrated using numerous other measures of brain anatomy and function. It cannot be excluded that the effects were modulated by amphetamine toxicity but effects of amphetamine use alone were not confirmed.

## Methods

### Clinical and demographic subject characteristics

Forty individuals with a history of amphetamine dependence (31 men, 9 women) were included in the study. The patients mean age was 38.5 years (st dev 9.1, range 21 - 55). Subjects with amphetamine dependence were recruited at Addiction Centre Stockholm, Sweden (Beroendecentrum). The majority of the patients (n = 35) were contacted as participants from previous treatment studies at the clinic. The remaining patients (n = 5) were contacted via advertisement or by word of mouth. Patients interested in participation contacted the study nurse and were then screened for eligibility. Inclusion criteria for the study were: a) men and women between the ages 20- 50 years; b) DSM IV diagnosis of amphetamine dependence; b) drug free for a minimum of 2 weeks and maximum 6 months prior to the scan. Exclusion criteria were: a) DSM IV diagnosis for any other substance dependence (except nicotine); b) DSM IV diagnosis for any major psychiatric disorder (e.g. mood disorders); c) any serious physical ailment (e.g. heart diseases or diseases that may affect brain function); d) HIV positive; e) presence of any metal objects in the body. Six of the patients were prescribed selective serotonin reuptake inhibitors (SSRIs). Two were prescribed tryptizol, and two were prescribed naltrexone.

Forty healthy control subjects (25 men, 15 women) who had previously participated in the Human Brain Informatics Project (HUBIN), Karolinska Institutet [32,33], participated. The mean age of the healthy control subjects was 42.7 (st dev 8.6), range 22 - 53 years. Exclusion criteria for the healthy subjects were a) any psychiatric diagnosis or treatment; b) psychotic disorder in first degree relatives; c) any physical disease or ailment that might affect brain function; d) presence of any metal objects in the body. All subjects were offered a free medical examination and 500 SEK (approx. 50 Euros) for their participation in the study. The study was approved by the regional ethics committee of Karolinska Hospital, Stockholm, Sweden. All participants had given written consent to participate in the study, and had the opportunity to withdraw at any point.

Demographic characteristics of the amphetamine patients and healthy control subjects are presented in Table 1.

**Table 1:** Demographical Characteristics.

		patients	controls
Gender:	Male	77.5%	66%
	Female	22.5%	34%
Education:	Common school	50.0%	-
	High school	45.0%	-
	University	5.0%	-
	Years in school	10.5 ± 2.6 years	14.3 ± 2.7 years
Employment:	Full-time	35.0%	95%^2^
	Part-time	15.0%	-
	Pension/Sick-leave	15.0%	-
	Unemployment	30.0%	5%
Civil status:	Married (or equal)	30.0%	71%
	Single-divorced	47.5%	13%
	Single-never married	22.5%	16%

^1^One subject only reported gender; one did not report years of education; one did not report either years of education or highest grade achieved.

^2 ^Controls were only asked if they were employed or not.

Demographic characteristics of the patients and controls. Completers: patients = 40, controls = 39

### Clinical Assessment

The patients in the present study fulfilled the diagnosis of amphetamine dependence based on the DSM-IV criteria. In addition to the DSM interview, patient drug usage patterns were measured via the Addiction Severity Interview (ASI), a structured interview which maps the severity and duration of dependence across seven sub-scales [34]. The Timeline Follow-back method, an interview technique used to register self-reported alcohol and drug use [35], corroborated the ASI results by inquiring about drug use history over the last 30 days. The interviews confirmed that the patients were D/L-amphetamine users. The average length of use was 13 years of regular usage (>4 days per week). Consumption ranging between 1-2 grams per occasion (average consumption of 6 grams per week). These amounts are typical for chronic amphetamine dependent individuals treated at the clinic.

Current usage was confirmed via urine toxicology tests screened for central stimulating amines, opiates, cocaine metabolite (benzoylecgonine), cannabis, dextroprophiphen, and benzodiazepines. Results from urine toxicology tests leading up until inclusion in the study demonstrated that all but one of the patients used amphetamine, with the remaining patient having used methamphetamine on several occasions. In order to insure that patients were indeed drug-free at the time of study, the patients had to provide two consecutive negative urine samples. The patients in the study were non-treatment seeking and it took a mean of 7.6 weeks to have them medically stabilized. Table 2 summarizes patient substance abuse characteristics.

**Table 2:** Patient Substance Abuse Characteristics.

	Mean (sd)
Age of onset amphetamine abuse	21.4 (7.4)
Amphetamine usage (y.i.l.)	13.3 (8.6)
Intravenous usage (73.8%)^1 ^(y.i.l.)	8.8 (9.6)
Amphetamine abstinent weeks before MR-scan	7.6 (10.2)
Age of onset any alcohol^2^	16.2 (4.0)
Any alcohol use (y.i.l.)	9.4 (9.8)
Age of onset heavy alcohol^3^	16.6 (4.6)
Heavy alcohol use (y.i.l.)	8.6 (10.2) y.i.l
Nicotine use	90.0%
Heroin (y.i.l.)	0.1 (0.4)
Pain relievers (opiates or equivalent) (y.i.l.)	1.1 (4.4)
Benzedrine (y.i.l.)	1.4 (4.5)
Cocaine (y.i.l.)	0.6 (1.8)
Cannabis (y.i.l.)	5.1 (6.5)
Hallucinogenics (y.i.l.)	0.5 (1.7)
Inhalants (y.i.l.)	0.3 (1.3)
Multiple drug use (y.i.l.)	6.6 (9.5)

Abbreviation: y.i.l. = years in life.

^1 ^73.8% The ASI does not ask which drug was used intravenously. The remaining ASI questions, as well as patient histories, suggest that for the current sample the intravenous usage was primarily amphetamine.

^2^A regular use of alcohol (less than 4 (for women) or 5 (for men) drinks per occasion). Four patients responded that they never began to consume any amount of alcohol regularly.

^3^A regular heavy use of alcohol (More than 4 (for women) or 5 (for men) drinks per occasion) Five patients responded that they never began to consume alcohol heavily.

Clinical characteristics obtained from ASI in patients with amphetamine dependence. Completers = 40.

Patients had been recruited based on not having a diagnosis of alcohol dependence. Nonetheless, the ASI found that 31 of the 40 patients met ASI criteria for heavy alcohol consumption (more than 4 (for women) or 5 (for men) drinks per occasion; 1 drink represents 12 grams of ethanol) for at least one year, and often several years, of their amphetamine abuse career. Figure 1 shows a plot of years amphetamine v.s. years heavy alcohol consumption.

Testing for infection with hepatitis C (HCV) was added to the study protocol after 6 patients had been included. Testing was conducted by referring patients to a medical laboratory. The laboratory tested for HCV via standard assay. Results were returned to our clinic. Of the 34 subjects referred for testing, 17 (50%) were HCV positive, 13 (38%) were negative, and 4 (12%) did not consent to the test.

Screening for HIV, an exclusion criteria, was featured from the beginning of the study and conducted via verbal inquiry. No screening was made for a diagnosis of attention-deficit hyperactivity disorder.

Control subjects had previously been characterized via the structured clinical interview for DSM-III-R (SCID-I) [36,37] administered by a experienced psychiatrist (EGJ) at one or two occasions between the years 1999 and 2008. SCID module E includes inquires into any use of illicit drugs and alcohol. Alcohol usage patterns in controls were further assessed by the Alcohol Use Disorders Inventory Test (AUDIT) [38,39]. These interviews include asking the subjects for their present alcohol use (consumption the last month) and their average consumption of alcohol during the month when they used alcohol most frequently on a lifetime basis. Control subjects were not asked to complete the ASI as they were not addicts.

The interviews were completed at least once by 39 and two times by 31 of the 40 subjects. At the first interview, conducted between 1999 and 2003, 38 control subjects completed the SCID interview and 37 the AUDIT. At the second interview, conducted between 2003 and 2008, 31 controls fulfilled the SCID interview and the AUDIT. The average total consumption of alcohol the month before the first and second interview was 309 g and 216 g, respectively. The mean AUDIT score from the first and second exam was 3.81 ± 3.04 and 3.13 ± 2.36, respectively. The difference in the two scores was not significant. A score in the range 3.13-3.81 is consistent with consumption of less than 4 drinks/week. The second round of interviews were given by the same researcher (EGJ).

None of the controls reported current heavy alcohol use or use of other illicit drugs, although one of the male subjects scored 9 on the AUDIT. Eleven of the controls (28%) used nicotine. Sixteen control subjects reported past use of marijuana, with 11 of these reporting less than 15 total usages. Seven claimed to have experimented with (used < 15 times) other illegal substances (i.e. cocaine, opiates, psychedelic mushrooms). One had taken anabolic steroids. These subjects were not excluded for two reasons. The limited exposure during such experimentation was not expected to alter the measure of interest, brain cortical thickness. Further, as limited experimentation is reflective of the general population, selecting for control subjects with no previous drug exposure could also introduce sampling bias.

### Image acquisition and processing

All participants were scanned using a 1.5 T General Electric Signa Exite MR system (Milwaukee, WI, USA) at the MRI-research centre, Karolinska Hospital, Stockholm, Sweden. Image acquisition included two 3D Spoiled Gradient Recalled (3D-SPGR) T1-weighted image series, with the following acquisition parameters applied: 124 contiguous 1.5 mm coronal slices, 35 degree flip angle, repetition time (TR) = 24 ms, echo time 6.0 ms, field of view (FOV) = 220 mm, acquisition matrix = 256 × 192.

All image series were inspected for gross pathology by a specialist in neuroradiology. Acquisitions with gross artifacts from excessive head motion or scanner instability were excluded from further analysis. Drug-free status at the time of scan was ascertained by urine toxicology tests.

Scan processing and statistical analysis was performed using the FreeSurfer image analysis suite, version 4. FreeSurfer is documented and freely available for download online http://surfer.nmr.mgh.harvard.edu/. All non-automated image processing was performed by a single, experienced image analyst. Technical details of the procedures are described in prior publications, including [40-44]. Briefly, the processing includes motion correction and averaging of multiple volumetric T1 weighted images. In 9 cases only one image series was used in subsequent analysis, either because one series was corrupted by head movement or artifacts, or because only one series was successfully retrieved from the scan. Motion correction was followed by removal of non-brain tissue using a hybrid watershed/surface deformation procedure [44], automated Talairach transformation, intensity normalization [45], tessellation of the grey matter white matter boundary, automated topology correction [43,46], and surface deformation following intensity gradients to optimally place the grey/white and grey/cerebrospinal fluid borders at the location where the greatest shift in intensity defines the transition to the other tissue class [40,47,48]. This method used both intensity and continuity information from the entire three dimensional MR volume to produce representations of cortical thickness, calculated as the closest distance from the grey/white boundary to the grey/CSF boundary at each vertex on the tessellated surface [48]. The maps produced were not restricted to the voxel resolution of the original data and thus are capable of detecting sub-millimeter differences between groups. Procedures for the measurement of cortical thickness have been validated against histological analysis [49] and manual measurements [50,51]. FreeSurfer morphometric procedures have been demonstrated to show good test-retest reliability across scanner manufacturers and across field strengths [52].

A template cortical surface was made by averaging the cortical surfaces of the subjects in the study. Standardized hemispheres from all participants were co-registered to this template using a non-linear surface deformation algorithm, where gyral folding patterns were the substrate of increased inter-subject alignment of homologous brain surface structures [42]. Surfaces were smoothed using a 10 mm full-width half maximum Gaussian kernel.

### Statistical analysis

All analysis followed a similar format. Cortical locations with significant group differences were found by contrast analysis of a general linear model fitted independently at each vertex in the aligned cortical thickness maps. Analysis was conducted using the FreeSurfer software suite as cited above. The model accounted for age and years of amphetamine usage. The output of the contrast analyses was visualized by creating brain images displaying the p-values as colors with intensity increasing on a logarithmic scale. Multiple comparison control was conducted by using the False Discovery Rate (FDR) [53,54] to determine alpha levels at which the expected proportion of false positives would be less than 5%. Visual inspection of these images identified regions containing significant group differences.

Followup analysis restricted itself to patients with less than 10 years of heavy alcohol use (*A*_<10_). The cutoff at ten or more years of heavy alcohol use was motivated as follows. Reductions in total cortical grey matter volume, particularly in the prefrontal and frontal grey matter, have been associated with the amount of time during which alcohol dependence symptoms were experienced [28], suggesting that alcohol-related brain tissue loss depends on length of use. Further, while our patients showed a sample correlation between years of amphetamine abuse and years of heavy alcohol consumption (Pearson's *r *= 0.49, *p *= 0.001), inspection of a plot of amphetamine vs alcohol usage (Figure 1) suggested that this correlation was mostly due to the 14 subjects with ten or more years of heavy alcohol consumption. Further correlation analysis supported this cutoff, with the measured sample correlation for these subjects remaining strong (*r *= 0.77, *p *= 0.001) while for patients with less than ten years of heavy alcohol consumption (n = 26) it was non-significant (*r *= -0.31, *p *= 0.13). We note that similar arguments would also support a cutoff of nine or more years. The final decision to use a threshold of ten years was made to give balanced subject numbers for the comparison of short- vs long-term amphetamine users (contrast e).

## Abbreviations

ASI: Addiction severity interview; AUDIT: Alcohol use disorders inventory test; FDR: False discovery rate; HCV: Hepatitis C; HIV: Human immunodeficiency virus; SCID: Structured clinical interview for DSM-III-R; WMH: White matter hyperintensities.

## Authors' contributions

GL drafted the final manuscript, performed literature search, and designed the data analysis. PSB drafted the preliminary manuscript, performed the scan workups, and the data analysis. AH, NJ-L, and JF participated in study design, patient recruitment, and interpretation of the results. IA participated in the study design and contributed scientific insight and content to the final manuscript. All authors have read and approved the final manuscript.
